# 

**Published:** 1858-02

**Authors:** 


We have on hand such an amount of original matter, that we were compelled to leave out a review of West cn Diseases of Women, from the pen of one of our most able collaborators. We were anxious to present to our readers in the present number, the article on microscopic examination of blood stains, by Robin, which we think is a paper of great practical utility. Several books stand over to be noticed in our next number. F.

*A New Medical School at Nashville.*—We learn that the brilliant success of the medical department of the University has prompted a new enterprise in the way of a Medical School at Nashville. We understand that it is to be under the patronage of the Methodist denomination.—*Cincinnati Lancet & Observer.*

